# Biological mechanisms matter in contemporary wildlife conservation

**DOI:** 10.1016/j.isci.2023.106192

**Published:** 2023-02-14

**Authors:** Steven J. Cooke, Christine L. Madliger, Robert J. Lennox, Julian D. Olden, Erika J. Eliason, Rebecca L. Cramp, Andrea Fuller, Craig E. Franklin, Frank Seebacher

**Affiliations:** 1 Fish Ecology and Conservation Physiology Laboratory, Department of Biology and Institute of Environmental and Interdisciplinary Science, Carleton University, 1125 Colonel By Dr., Ottawa, ON K1S 5B6, Canada; 2 Department of Biology, Algoma University, 1520 Queen St. East, Sault Ste. Marie, ON P6A 2G4, Canada; 3 Norwegian Research Centre (NORCE), Laboratory for Freshwater Ecology and Inland Fisheries, 5008 Bergen, Norway; 4 School of Aquatic and Fishery Sciences, University of Washington, Seattle, WA 98195-5020, USA; 5 Department of Ecology, Evolution and Marine Biology, University of California, Santa Barbara, Santa Barbara, CA 93106, USA; 6 School of Biological Sciences, University of Queensland, Brisbane, QLD 4072, Australia; 7 Brain Function Research Group, School of Physiology, Faculty of Health Sciences, University of the Witwatersrand, Parktown 2193, South Africa; 8 School of Life and Environmental Sciences A08, University of Sydney, Sydney, NSW 2006, Australia

**Keywords:** Biological sciences, Zoology, Evolutionary biology

## Abstract

Given limited resources for wildlife conservation paired with an urgency to halt declines and rebuild populations, it is imperative that management actions are tactical and effective. Mechanisms are about how a system works and can inform threat identification and mitigation such that conservation actions that work can be identified. Here, we call for a more mechanistic approach to wildlife conservation and management where behavioral and physiological tools and knowledge are used to characterize drivers of decline, identify environmental thresholds, reveal strategies that would restore populations, and prioritize conservation actions. With a growing toolbox for doing mechanistic conservation research as well as a suite of decision-support tools (e.g., mechanistic models), the time is now to fully embrace the concept that mechanisms matter in conservation ensuring that management actions are tactical and focus on actions that have the potential to directly benefit and restore wildlife populations.

## Graphical abstract

**Figure:**
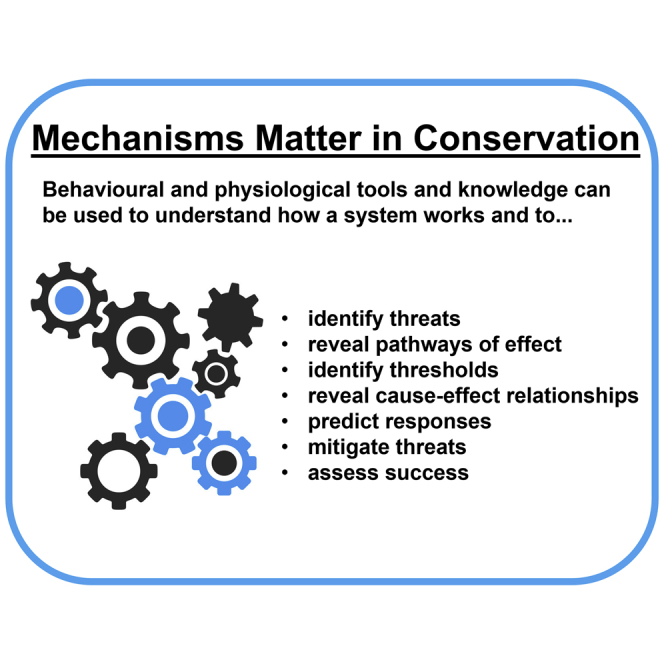

## Introduction

Conservation managers and practitioners use diverse forms of evidence, including empirical studies, evidence syntheses, experiential understanding, and indigenous and stakeholder knowledge^1^^,^^2^ to make decisions intended to protect or restore biodiversity.^3^ Although biodiversity inherently encompasses all levels of biological organization—from the molecule to the biome^4^—the vast majority of conservation decisions and actions are informed from evidence collected at the population level.^5^^,^^6^ Superficially, this conservation paradigm is appropriate in that populations represent somewhat discrete (often spatially and genetically^7^) and logical units for both assessing trends in abundance and guiding management in a strategic manner.^8^ From a practical perspective, many wildlife populations can be assessed such that trend-through-time data yield useful information on population trajectories,^9^ which serve as the basis for threat assessments (e.g., the International Union for Conservation of Nature [IUCN] Red Listing and associated regional analogues;^10^). Yet, data on population status may be meaningless without a robust understanding of the mechanisms driving demography (i.e., vital rates;^11^). In the absence of a mechanistic dimension (i.e., establishing causation), it is difficult to apply targeted conservation actions that are effective and focus on the appropriate mechanistic levers (i.e., cause-effect relationships) that are regulating population growth or persistence.^12^

In this paper, we argue that mechanisms matter to conservation. We posit that if conservation scientists, managers, and practitioners were to seek and incorporate more mechanistic understanding of biological organization, conservation decisions would be more effective in protecting and restoring populations and indeed all forms of biodiversity. We first define what we mean by “mechanisms”. Next, we discuss why mechanisms matter in the context of connecting wildlife to their environment, identifying conservation problems, and generating conservation solutions. We then discuss mechanistic approaches to wildlife research (spanning experimental studies to modeling) relevant to conservation. Using diverse case studies, we highlight where mechanistic approaches to understand species declines have been applied and conclude with a candid assessment of what is needed to make conservation more mechanistic and, ultimately, more successful. We recognize that mechanisms are as relevant to plants and other taxa as they are to animals, yet for the purpose of this paper we restrict our discussions to animals (herein wildlife). The concept that mechanisms matter in conservation is not entirely new,^13^^,^^14^^,^^15^^,^^16^^,^^17^ but despite previous efforts to mainstream this idea, the approach has yet to be fully embraced. Moreover, most of the previous treatments did not consider mechanisms the same way as we do here. Our treatment is more rooted in organismal physiology and animal-environment interactions (see next section), which we argue is particularly salient and timely given the current biodiversity and climate crises. Here we center the concept of mechanisms being important for conservation and provide a clear and direct narrative regarding the value in doing so while providing explicit examples of how that can be achieved.

## What do we mean by mechanisms?

There are many formal definitions of mechanisms (see reviews^18^^,^^19^), and one that works well with biological systems and conservation is from Bechtel and Abrahamsen^20^: “A mechanism is a structure performing a function in virtue of its component (causally interacting) parts, component operations, and their organization. The orchestrated functioning of the mechanism is responsible for one or more phenomena.” Machamer et al.^21^ defines mechanisms as “entities and activities organized such that they are productive of regular changes from start or set-up to finish or termination conditions.” In ecology, we use mechanisms to help explain how a phenomenon occurred/occurs as a result of the causal chain(s) of its integrated parts (see Connolly et al.^22^ for a detailed discussion of mechanisms and processes in ecology). For example, the past experiences of the organisms (across various temporal scales) and ecological interactions may be explained through the dissection of a series of events, the interplay of processes and causal chains. In short, mechanisms are about how a system works^19^ with the assumption that they are relevant to understanding and establishing causal relationships that may transcend scales (e.g., levels of biological organization^23^).

Individual animals are themselves complex systems as are the populations they are part of, the assemblages they belong to, and the ecosystems in which they are embedded. At the level of the individual, biological mechanisms that span physiology and behavior (and are underpinned by genetics, evolutionary history, and selection^24^^,^^25^) are the first point of contact between organism and environment, and the mechanism-environment interaction influences organismal performance and fitness (including survival^26^). Animals are thereby linked to their environment so that, when environmental conditions are suboptimal, there can be consequences that, in extreme cases, may be lethal (as elegantly outlined in the Fry Paradigm; see Kerr^27^). Behavior and physiology are inherently connected in that physiology constrains and enables behavior, whereas behaviors (e.g., feeding, habitat selection, movement) have physiological consequences.^15^^,^^28^

**Figure 1:**
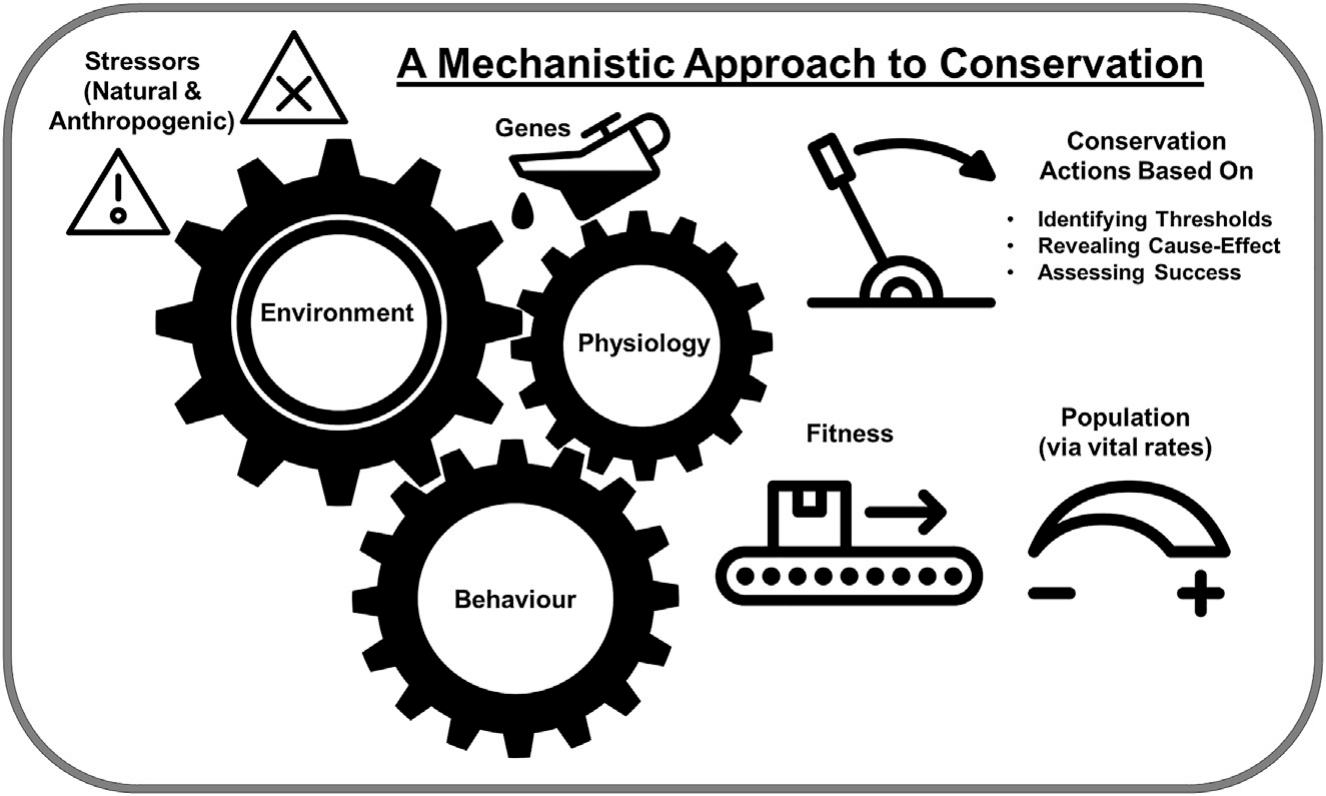
A mechanistic approach to conservation Simple conceptual diagram visualizing ways in which environmental stressors (natural and anthropogenic) act on individuals (mediated by genes that underpin the connections between organism and environment) via behavioral and physiological pathways (often at their intersection) that have the potential to influence fitness at the level of the individual and contribute to population vital rates. Conservation actions represent levers that can be applied in various ways (e.g., to remove a stressor or to otherwise compensate for it by directly mediating behavior, physiology, and fitness) with a goal of altering population vital rates. Conservation actions are based on mechanistic knowledge related to thresholds, cause-effect relationships, and the assessment of conservation success.

Mechanistic relationships and interdependencies evolve to provide animals the means with which to cope with change and restore homeostasis when encountering challenges.^29^ Given the interplay between environmental conditions and fitness, this mechanistic dimension scales up to the population and influences demography.^26^^,^^29^ Mechanisms can also extend across domains. For example, conservation issues almost always have a human dimension, and failure to consider such drivers or impacts would be problematic.^30^ Given the manifold benefits that humans derive from biodiversity, loss of biodiversity can have consequences that extend beyond reductions in, say, wildlife fitness. Understanding mechanisms and mechanistic pathways reveals causal relationships that can be pursued in conservation.^12^ For the purpose of this paper, we focus on the mechanistic intersection of physiology, behavior, and environment with considerations of consequences that extend from the individual to higher levels of biological organization (Figure 1). We take a pragmatic approach to applying mechanisms to conservation challenges, acknowledging that some scenarios will benefit from considering the interconnectedness of multiple aspects of physiology and behavior, while others may only require measurement of one mechanistic component.

## Why do mechanisms matter?

### Mechanisms connect animals to their environment

Animal performance is intricately linked to the surrounding environment—for ectotherms^31^ and endotherms.^32^ Individual physiological responses and capacities to respond to environmental drivers determine performance, ecological relationships, and the persistence of populations and species across space and time.^26^^,^^33^ It is physiology that acts as a filter between the environment and fitness-related functions and provides the mechanism that supports and underpins our understanding of the ecology of a species and connects animals to their environment.^26^^,^^34^ Changes in environmental drivers and mechanisms are intertwined; identifying how causal factors lead to species decline must be elucidated for the purposes of conservation.^26^^,^^35^ We acknowledge that there are other types of mechanisms relevant to conservation, but these are at a higher organizational level than the physiological mechanisms which are the focus of this paper. For example, understanding species interactions (e.g., predator-prey,^36^ plant-herbivore,^37^ and host-pathogen interactions^38^) has the potential to reveal mechanisms that are relevant to conservation. However, these interactions are one step removed in complexity from the direct interaction between environment and physiology, although not independent from environmental impacts on physiology (e.g., physiology underpins locomotion, which underpins predator-prey interactions).

Environmental change and conservation interventions both act on animal biology. Abiotic environmental changes, such as changes in temperature, typically elicit behavioral and physiological responses.^39^ Changes in the biotic environment, such as interactions between individuals or their society, can affect physiology via endocrine-mediated processes. For example, social interactions can induce changes in metabolic function.^40^^,^^41^ Conservation measures target biological processes by preserving or restoring particular habitats or aspects of the physical environment to improve individual fitness (reproductive outcomes and survival), assure population persistence, and maintain biodiversity.^42^ For example, reforestation or pollution remediation alters the physical and chemical environments with beneficial outcomes for resident species.^43^^,^^44^ However, unlike Newton’s Third Law of action and reaction, the environmental or conservation inputs (action) do not necessarily produce a proportional biological output (reaction). Even simple thermodynamic effects of changes in acute temperature produce a non-linear output in physiological reaction rates. These relationships are complicated by evolutionary processes of adaptation and plasticity.

Adaptation, genetic drift, or gene flow can alter genotypes and phenotypes across multiple generations.^45^ Genetic differences may thereby match species and populations to their environment and increase performance and fitness. For example, genetic divergence was the likely mechanism that conferred increased heat tolerance on urban ants (*Temnothorax curvispinosus*) compared to rural populations.^46^ Insights into genetic divergence between populations and the link between genotype and physiological phenotype provide essential information for conservation decisions. In the case of ants above, the urban heat effect^47^ would be offset by the greater heat tolerance of urban populations therefore lessening the urgency to implement conservation actions. Genetic diversity among populations can be important if it determined the sensitivity of individuals to environmental variability and their capacity to respond to change, and population genetics thereby play a major role in conservation.^48^^,^^49^ However, particular genotypes are by no means fixed to perform well only within a narrow range of environments within which they have evolved. An environmental change may present a novel selective environment eliciting further genetic changes.^50^ At a more rapid timescale and superimposed on these genetic changes are epigenetic modification of gametes in parents that transmit environmentally induced modifications of gene expression pattern to the next generation.^51^ In addition, environmental signals experienced within individuals early in development (developmental plasticity) or in adult organisms (reversible acclimation) can alter phenotypes in response to short-term environmental fluctuations.^52^^,^^53^ Epigenetic effects in response to environmental signals are transmitted via molecular changes to DNA and proteins by DNA and histone methylation and acetylation of proteins.^54^^,^^55^^,^^56^^,^^57^ Like a Fourier series in physics where oscillations with different periodicity are superimposed onto each other to produce complex responses, combinations of genetic and epigenetic change result in complex phenotypes that can respond to environmental change at different timescales. Understanding these dynamics is important for conservation because they define how quickly and at what magnitude individuals, populations, and species can respond to environmental change and potentially compensate for its effects. For example, an effective epigenetic response can induce plasticity that buffers physiological processes from an external change in temperature,^58^ which would render the organism a low conservation priority in this particular case. On the other hand, in cases where plasticity is absent or ineffective a temperature increase may have strong detrimental effects so that immediate conservation action is necessary.^59^ However, this complexity of mechanisms and temporal resolution also means that phenotypic responses are not easily predictable, thus pointing to the need for experimental physiological data to resolve these transient responses.

Intuitively, plastic responses are beneficial because they can offset potentially negative impacts of environmental change on physiological processes.^60^ However, disadvantages may arise when there is a mismatch between the environmentally induced phenotype and the actual environment experienced.^61^ In addition, beneficial plastic responses, such as compensation of swimming performance in fish for changing temperatures, may trade off against concurrent costs such as energetic cost of locomotion.^62^ Greater energetic costs may cause an allocation trade-off within individuals so that energy is preferentially allocated to, for example, growth at the expense of reproduction.^62^ It is crucial that evaluations and predictions of environmental impacts on natural systems, and conservation measures following these assessments, explicitly account for the temporal and mechanistic dimensions outlined above.^34^^,^^63^^,^^64^

Species distribution models (SDMs) are a useful tool to assess biodiversity in different geographic regions. Often, these models incorporate climate data to predict the potential distribution of species under current conditions and different scenarios of climate change.^65^^,^^66^ At their most basic, SDMs (e.g., climate envelope models) use information about environmental conditions prevalent in current geographical ranges of species to model suitable geographical areas following an environmental change such as climate change.^67^ Predictions by SDMs could be improved by incorporating key physiological processes,^68^ demographic processes,^69^ and the genetically and epigenetically modified response dynamics of these processes.^34^ Accordingly, biophysical models add independently sourced data on environmental sensitivities of physiological processes to model potential species distributions in changing environments.^70^ The most recent models explicitly include estimates of plasticity and adaptation to estimate species distributions and their vulnerability to environmental (climate) change.^71^^,^^72^ Ultimately, the more information a model contains, the better its capacity to predict changing species distribution. The latter class of models that include plasticity and adaptation are still quite rare but are likely to provide the best estimates^71^ particularly for physiological responses that are well known to be regulated epigenetically. Also noteworthy are recent developments in genomic offset estimates (prediction of maladaptation to future climate) that can be used alongside SDMs.^73^

Key physiological processes may vary between species, although there is some agreement of processes that are important in a conservation context.^63^ Disruption of physiological processes that are essential for whole organism function (e.g., energy metabolism) is likely to compromise fitness, and these processes therefore represent a focus for conservation. Therefore, rather than focusing on downstream consequences of environmental change, it may be fruitful to consider the underlying regulatory pathways. For example, endocrine pathways are at the interface between the environment and organism behavior and function, and anthropogenically induced endocrine disruption can have a broad range of downstream effects.^74^^,^^75^ A focus on the hormone rather than on its effect on individuals may therefore be an effective approach to identify and target specific remediation strategies.

### Mechanisms can be used to elucidate problems

Environmental change introduces a myriad of challenges to animals in the wild, and a major tenet of conservation biology is to identify and resolve multiple stressors that are limiting the productivity of animal populations.^64^^,^^76^ Major stressors that interact mechanistically with individuals and cause a physiological and behavioral response can be isolated by experimentation. Mitigating the stressor or employing other conservation approaches that minimize the behavioral and physiological consequences on the animal can form the basis of effective management. Such interventions are contingent on an understanding of the mechanistic responses of animals to environmental change and on access to a conservation toolbox that assists scientists and practitioners in untangling the often-complex effects of multiple stressors. Where multiple stressors are operating on individuals, identifying problems and revealing mechanistic relationships between a single stressor and a response at the individual or sub-individual scale are more challenging, particularly where there are synergistic or antagonistic relationships between/among stressors.^77^ Isolating stressors may include consideration of why and how animals die based on additive vs compensatory mortality frameworks or component-cause models. Failure to isolate mechanisms causing individual disturbance and impacting population demographics has been a major challenge for biodiversity conservation in a multi-stressor world, and conservation can benefit from experiments and null model testing.^78^

Working on realistic conservation timescales to identify stressors and mechanisms in the field when conservation action is needed may not always be possible and may not align with the timelines available for funding to address conservation issues, which are chronically resource limited (especially time). Laboratory manipulations or mesocosm experiments can identify mechanisms more rapidly and scale solutions to the field. In the future, simulations may be helpful to isolate mechanisms using probabilistic algorithms based on a comprehensive understanding of the action-reaction relationships that exist between animals and their environment. Operationalizing mechanisms as conservation tools requires frameworks for rapid assessment and off-ramps for general action to be taken when mechanisms are difficult to disentangle due to multiple stressors. Rapid assessment frameworks are necessary given that it is possible to get lost in the mechanisms. We are advocates of fundamental research, so engaging in detailed work is always intellectually useful; but for mission-oriented conservation research, it is important to focus on mechanisms and pathways relevant to a given threat, issue, or problem. For example, in the salmon case study below, simple reflex impairment assessments have been useful for assessing fish condition in the field after fisheries interactions and providing fishers and managers with tools to quantify and reduce bycatch mortality.

### Mechanisms can be used to identify solutions

Physiological and behavioral mechanisms have become increasingly valued for detecting causes of disturbances to wildlife^79^^,^^80^^,^^81^; however, their combined use in delineating conservation solutions is still gaining momentum.^35^ By identifying or confirming the environmental stressor responsible for disturbance and how it influences organismal function, integrated mechanistic approaches can formulate conservation solutions to address the underlying problem.^82^^,^^83^ For example, recognizing that there is a mismatch in the thermal tolerance of some amphibian hosts and *Batrachochytrium dendrobatidis* (Bd; the fungal pathogen that causes chytridiomycosis), the creation of elevated temperature microhabitats where behavioral thermoregulation may assist in the management of pathogen infection intensity could be an effective conservation undertaking for some species.^84^ An integrative behavioral and physiological approach can also facilitate improved monitoring of disturbances, thereby enabling faster detections of problems and providing more time to take conservation action. For instance, thermal physiology and behavioral measurements together lead to more informed ways to monitor *Pseudogymnoascus destructans* infection (i.e., white nose syndrome) in wild bat colonies.^85^ Further, morphology, physiology, and behavior interact to permit or constrain performance within environmental contexts;^86^ thus, considering this interconnectedness can provide information on critical habitat requirements and future vulnerability (e.g., to climate change^87^^,^^88^^,^^89^). Studying these form-function relationships can therefore determine areas for protection based on species’ tolerances^83^^,^^90^ or identify potential stocking or translocation locations incorporated, for example, into programs for imperiled fishes.^91^^,^^92^ Considering the connections between behavior and physiology can also improve post-release survival following reintroductions, for example by informing predator-training programs and soft-release protocols.^93^

Simultaneously monitoring behavioral and physiological traits has improved identification of reproductive readiness^79^^,^^94^ and overall welfare in captive programs (e.g., tigers^95^; elephants^96^). In terms of *in situ* conservation applications, determining the physiological underpinnings of migratory behavior can allow forecasting of migratory timing, allowing managers to put temporal and regional restrictions in place to minimize disturbance.^97^ Similarly, by identifying the most energetically demanding parts of migratory routes, linking physiology and migratory behaviors can impart valuable knowledge for protecting key stopover habitats or implementing supplementation programs.^97^ Finally, understanding the physiological and behavioral responses of organisms to chemical and physical characteristics in their environment is leading to innovation in invasive species control (e.g., sea lamprey [*Petromyzon marinus*]^98^) and the design of human infrastructure to keep native species from interacting with lights, dams, turbines, and fishing nets, among others.^16^^,^^99^

## Case studies that demonstrate a mechanistic approach

We selected several case studies that highlight how a mechanistic approach has been applied to a variety of wildlife taxa (e.g., fish, amphibians, mammals) and issues (e.g., multiple stressors, disease, invasive species, climate change). Moreover, the case studies rely on various empirical and modeling methods that span physiology and behavior with some consideration of ecological mechanisms.

### Pacific salmon management

**Figure 2:**
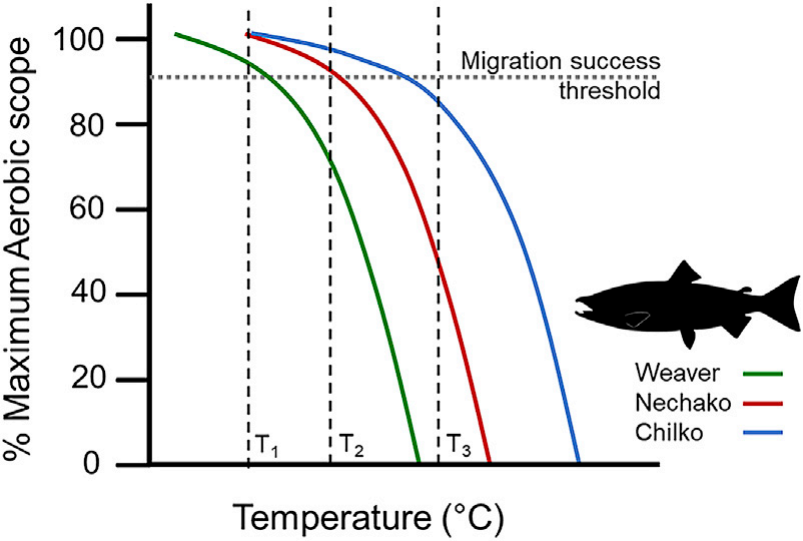
Pacific salmon performance A conceptual figure showing the percentage of maximum aerobic scope as a function of temperature (cool to warm) is shown for three Fraser River, BC, Canada sockeye salmon populations: Weaver, Nechako and Chilko. Although it is unknown exactly how much aerobic scope is necessary to successfully migrate upstream, 90% is indicated as a likely threshold. At cooler river temperatures (T1), all populations have near-maximal aerobic scope at near-optimal migration temperatures. As temperatures warm (T2), Weaver salmon are unlikely to reach their spawning grounds while Chilko and Nechako salmon are within their optimal temperature range for migration. If river temperatures continue to rise (T3), even Chilko salmon are unlikely to reach their spawning grounds. Tagging studies revealed that Weaver salmon abnormally entering the Fraser River early encountered warm water temperatures (e.g., T2 and T3) exceeding their optimal threshold for aerobic scope which could partially explain migration failure.^103^

The Fraser River of British Columbia (Canada) is home to sockeye salmon *Oncorhynchus nerka* which undertake iconic migrations from natal rivers to the high seas to feed and then back again to spawn. Because sockeye salmon are semelparous, failure to return to spawning grounds to reproduce means that lifetime fitness is zero. Although mortality occurs throughout all phases of the salmon life cycle, attention has predominantly focused on understanding adult upriver spawning migration given that in some years >90% of sockeye salmon that enter the river fail to make it to spawning grounds. Migration inherently connects behavior and physiology with environmental conditions such that adopting a mechanistic approach to understand reduced fitness is essential. Following several years of abnormal migration behavior (fish returning early) and exceedingly high rates of en route mortality, a large-scale mechanistic research program was initiated.^100^ Both observational and experimental research revealed that early migrants were more reproductively advanced and ill-prepared for osmoregulatory transition upon their entry into fresh water.^101^ Moreover, genomic studies revealed that early migrants were immunocompromised.^102^ Because fish were entering the river early, they were exposed to water temperatures that were much higher than historic norms, which exacerbated disease progression and energy depletion.^101^ Swimming performance and respirometry studies revealed population-specific variation in thermal optima, and, for some populations, the warmer temperatures exceeded their critical maxima leading to the collapse of metabolic and cardiac scope ending in death (Figure 2;^59^). Although some questions still remain, it is apparent from this body of research that water temperatures are major drivers of mortality for early migrants. Salmon also encounter fisheries, and there are instances when fish are released (e.g., regulations require release of given species as part of selecting fishing policies or conservation ethics of an angler or indigenous fisher). Simple reflex indicators have been developed that provide rapid (and free) information on the vitality of fish that is predictive of survival (i.e., low reflex impairment = high survival, high reflex impairment = low survival^104^). After these tools were validated,^105^ they provided fishers and managers with new tools to understand mechanisms impacting captured salmon and to adjust fishing practices to reduce impacts in real time. Although there was initial skepticism by fisheries managers about undertaking mechanistic research,^106^ the creation of clear cause-effect pathways combined with the ability to explain population-specific mortality patterns has led to these approaches being embraced and incorporated into fisheries planning.^107^ Moreover, the reflex assessments can be done by fishers, so they can adjust their fishing practices in real time.

### Amphibian disease

Amphibians are at the forefront of the global biodiversity crisis due in large part to the emergence of the novel fungal skin disease chytridiomycosis, which has been linked to the decline or extinction of more than 500 amphibian species globally.^108^ Mechanism is at the heart of understanding chytridiomycosis: its origins; how, why, when, and where it affects hosts; how individuals, populations, or species respond to infection; how ongoing or increasing environmental change alters disease risks for other populations and species; and how we manage disease risks to facilitate successful conservation. Like many diseases, the prevalence of chytridiomycosis is highly environment dependent as a result of impacts on both host physiological function and the thermal biology of the fungal pathogen. Anthropogenic environmental change likely contributed both to the emergence of the hypervirulent fungal lineage responsible for the disease chytridiomycosis and its ongoing impacts on global amphibian populations.^109^ A mechanistic understanding of what constitutes optimal environmental conditions for pathogen survival and disease development in hosts has been pivotal in allowing researchers and conservation managers to model and predict the emergence^110^ and spread of the pathogen under current and future climates,^111^^,^^112^^,^^113^ the likely severity of disease,^114^ and the seasonal dependence of outbreaks.^115^ Elucidating the mechanisms underpinning host susceptibility to the pathogen and the pathology of the disease has revealed why some species and life stages are more susceptible to the pathogen.^116^^,^^117^^,^^118^^,^^119^ Equally, an understanding of mechanism has been essential to interpret important host behavioral responses to infection such as increased skin shedding^120^ and thermoregulatory changes^121^ to manage pathogen loads and learned avoidance of the pathogen.^122^ Importantly, an understanding of mechanism has been central to the development of effective treatments for infected frogs^117^^,^^123^^,^^124^ and to the management of chytridiomycosis in conservation-significant amphibian populations worldwide.^125^

### Invasive species control

Invasive species are responsible for profound, negative effects on biodiversity,^126^ ecosystem functioning and services,^127^ human health^128^ and welfare,^129^ and the economy.^130^ Developing effective management strategies and policy to avoid or reduce the impacts of invasive species is of paramount importance. Mechanistic models represent indispensable tools for invasive species management as they allow scientists to estimate key vital rates such as spread rate, simulate the potential effects of invasive species, and explore implications of various control or eradication strategies.^131^ These models can couple the temporal and/or spatial dynamics of invasive populations with the ability to simulate and evaluate management actions in terms of specified outcomes.^132^ For example, Grechi et al.^133^ integrated a multi-objective decision framework with mechanistic population growth models to predict the population dynamics and optimal management of buffelgrass (*Pennisetum ciliare*), a commercially valuable invasive species, in Australia.

Mechanistic models based on physiological data seek to capture system dynamics at larger spatiotemporal scales compared to those at which empirical data are typically available and field experimentation is possible, with the desire to better support management decisions.^134^ Recently, new methods have been suggested to combine SDMs - which correlate species occurrence or abundance with environmental predictors—with physiological estimates of performance—such as temperature thresholds to delineate the range of environmental conditions within which the species can survive—to improve forecasts of species distributions when extrapolating to novel climate scenarios.^70^ For example, the utility of physiology-SDMs has been demonstrated for predicting invasion risk of non-native marine species in the Mediterranean,^135^ and if linked with economic analyses, mechanistic models can explore the costs of management and identify strategies that strike an optimal trade-off between management objectives such as suppression and cost.^136^^,^^137^ The advantages of mechanistic SDMs, however, are tempered by the fact that species information, such as physiological data, is often limited and possibly biased, across taxa at large scales.^138^ This suggests that correlative SDMs may be more practical from an implementation perspective^139^ and in some cases may be favored because of comparable predictive power to mechanistic models.^140^

### Climate change and wildlife in warming and drying climates

Most predictive models of animal responses to climate change have focused on the direct effects of higher ambient temperatures. However, many terrestrial animals will have to contend with a concomitant reduction in food and water, which is likely to severely challenge their ability to maintain homeostasis.^141^ Determining how chronic sub-lethal effects of climate change affect fitness requires an understanding of the behavioral trade-offs that animals make as well as the physiological plasticity available to them to buffer the changes.^141^^,^^142^ For example, in the hot and arid Kalahari of southern Africa, there has been a decline in growth (body mass at 3-month) and survival rate in meerkat (*Suricata suricatta*) pups over time, which is associated with an increase in the daily maximum air temperature in the Kalahari over the last 20 years.^143^ One might expect the underlying mechanism to be a reduction in energy intake, either because prey is less available or because prey has lower energy content. Alternatively, there may be reduced foraging as a result of higher air temperatures constraining activity or an increase in energy expenditure associated with a greater foraging effort to locate prey. However, the reduction in the body mass gain of pups on hotter days was unlikely to reflect a change in energy balance as the pups were fed by adults, and feeding rate increased with increasing maximum air temperatures. Instead, the mechanism may be related to the effects of repeated dehydration on growth,^143^ an idea that requires further confirmation through both field and laboratory experimentation. Such mechanistic knowledge is crucial for determining whether appropriate conservation actions can be implemented for this species or whether the species distribution is likely to shrink with fewer suitable habitats available in future.

## Achieving mechanistic conservation

Using four case studies, we highlighted diverse ways in which a mechanistic approach to conservation can be useful for understanding and solving conservation problems. A consistent theme arising from all case studies was that multiple tools and approaches were useful for obtaining comprehensive understanding. Those approaches may involve work in the lab, field or, in silico (e.g., modeling). Conservation problems and issues are diverse with some requiring mechanistic understanding that may have a physiological, behavioral, or ecological basis, and there may be instances in which knowledge of one of those domains is needed and others where all three are necessary. We also acknowledge that for some conservation problems (e.g., spatial protection prioritization), mechanistic knowledge may not be required. Nonetheless, outcomes from mechanistic work such as identifying thresholds or developing cause-effect relationships are almost always helpful when setting conservation targets and ensuring that the correct conservation levers are being applied.

The status quo relies on monitoring populations and preserving habitat—both essential features of conservation. However, a mechanistic understanding of how animals interact with their environment will provide a clearer understanding of the match between habitat characteristics and animal needs, and at the same time, a better understanding of the impact of altered environmental drivers on animal populations (including their demography^144^). Physiological and behavioral knowledge can provide a first-principles understanding of how animals respond to landscape characteristics that contain much greater predictive power than descriptions of presence and absence. Physiology and behavior therefore must become an integral part in landscape assessment for conservation and integrated landscape use,^145^ in conjunction with evaluation of physical characteristics (e.g., hydromorphological and biophysical^146^) and species distribution modeling.^70^ Although much progress has been made in all of these domains in the last few decades, there remains need for long-term studies/monitoring to generate robust empirical data that fully reflect spatiotemporal dynamics and relationships that presumably govern animal-environment interactions.

There is a philosophical divide between “conservation ecology” and “physiology” that may constrain our ability to achieve mechanistic conservation. Most conservation practitioners manage wildlife populations such that a mechanistic focus may be regarded as irrelevant^12^). Yet, failure to understand mechanisms can lead to misallocated resources and ultimately may delay or prevent the recovery of a population. There is a need for more “cultural integration” among different branches of research that can also be achieved through broader training (that recognizes the value of mechanistic approaches in conservation) and the sharing of more success stories.^81^ Fortunately, there are more examples of success stories as exemplified by the case studies presented here.

Mechanistic approaches to complex applied problems have been central in the realm of neuroscience (over reductionist thinking^19^). Given that conservation problems are equally complex, mechanistic thinking has much to offer. From urban ecology^147^ to human-wildlife conflict^148^ to invasive species management,^131^ there have been numerous calls for making conservation more mechanistic. With a growing toolbox for doing mechanistic conservation research as well as a suite of decision-support tools (e.g., mechanistic models), the time is now to fully embrace the concept that mechanisms matter (where appropriate and relevant) in conservation. Approaching conservation from a mechanistic perspective (Figure 1) is not an academic exercise—it is about ensuring that management actions are tactical and focus on actions that have the potential to directly benefit and restore wildlife populations.
